# Cognitive Errors and Risks Associated with Provider Handoffs

**DOI:** 10.7759/cureus.3442

**Published:** 2018-10-12

**Authors:** Kenneth L Frye, Ademola Adewale, Carmen J Martinez Martinez, Clara Mora Montero

**Affiliations:** 1 Emergency Medicine, Florida Hospital, Orlando, USA

**Keywords:** cognitive errors, provider handoffs, safer sign out, emergency medicine, patient safety

## Abstract

The emergency department is a challenging environment to practice medicine, primarily due to the pace and logistics of practicing emergency medicine. Cognitive errors and provider handoffs can lead to poor patient outcomes. By acknowledging and addressing cognitive errors, including premature closure, anchoring, and diagnosis momentum, we can potentially improve patient care. Additionally, by completing thorough, yet efficient sign-outs, as per The American College of Emergency Physicians' (ACEP) “Safer Sign Out Protocol,” the chances of a poor outcome are further reduced. Below, a case of “migraine headache” is presented, highlighting cognitive errors and the risks associated with provider hand-offs in the emergency department.

## Introduction

Emergency medicine is considered a “cognitive” profession and, therefore, cognitive biases, as opposed to knowledge deficits, are the primary cause of errors. The dual-process theory is the dominant theory of human cognitive processes and involves two unique, interdependent, but overlapping systems. The first system uses primarily intuitive, unconscious mental shortcuts. These shortcuts are known as heuristics, which help us work through complex problems efficiently. The second system uses primarily analytical, conscious thought but is typically slow, deliberate, and requires effort [1]. Both of these systems help physicians provide high-quality patient care, but the speed and efficiency with which heuristics are performed contribute to the biases we see in emergency medicine.

Some of the common cognitive biases related to emergency medicine include premature closure, anchoring, and diagnosis momentum [2]. In addition to the cognitive biases associated with the case described below, the patient was also at risk for a poor outcome due to the nature of shift change and provider hand-offs. Communication errors have been found to be the cause of 70% of sentinel events and 84% of treatment delays. Of these, 62% are “continuum of care” issues associated with shift change [3]. Furthermore, up to 80% of serious medical errors involve miscommunication during handoffs [4], and up to 24% of emergency department (ED) malpractice claims involve inadequate handoffs [3].

Here we report a case of a rapidly progressive central nervous system (CNS) infection in the setting of multiple ED visits for migraines, highlighting cognitive errors.

## Case presentation

A 23-year-old female presented to the ED with six episodes of left-sided headaches in two and a half weeks. She reported photophobia, nausea, and vomiting and had an improvement in her symptoms with the use of medications such as prochlorperazine, diphenhydramine, and ketorolac. Despite multiple evaluations in the ED, a negative head computed tomography (CT), negative brain magnetic resonance imaging (MRI), and prescriptions for medications that were helpful in the ED, the patient kept returning with a headache. The patient denied any previous history of migraines prior to her first presentation but was given the label of "migraines" and was treated repeatedly with "migraine cocktails."

On this visit, the patient was seen about two hours before shift change and was reported to be alert, oriented, and with a Glasglow Coma Scale (GCS) of 15. The patient had received prochlorperazine, diphenhydramine, and ketorolac and was then signed out as: “a migraine, medicated, discharge pending improvement.” The patient was examined without the pre-handoff provider after sign-out and was noted to be drowsy, presumably due to prochlorperazine and diphenhydramine. However, after several hours of observation (three hours after medication administration) in the ED, with frequent examinations (patient persistently drowsy, GCS 10) the patient never returned to her neurologic baseline per her family at the bedside. While she had no focal deficits at this time, concerns from the family were raised as she began answering questions regarding her history incorrectly (per her family) and was displaying a change from her "normal" effect.

The patient’s initial diagnosis was a migraine headache and treatment for a migraine had been provided appropriately. When the patient was found to have altered mental status, as opposed to being drowsy from medication administration, the differential diagnosis was expanded. The expanded differential included medication effects (less likely due to the duration of symptoms) and acute intracranial processes such as bleeding, masses, and infections.

**Figure 1:**
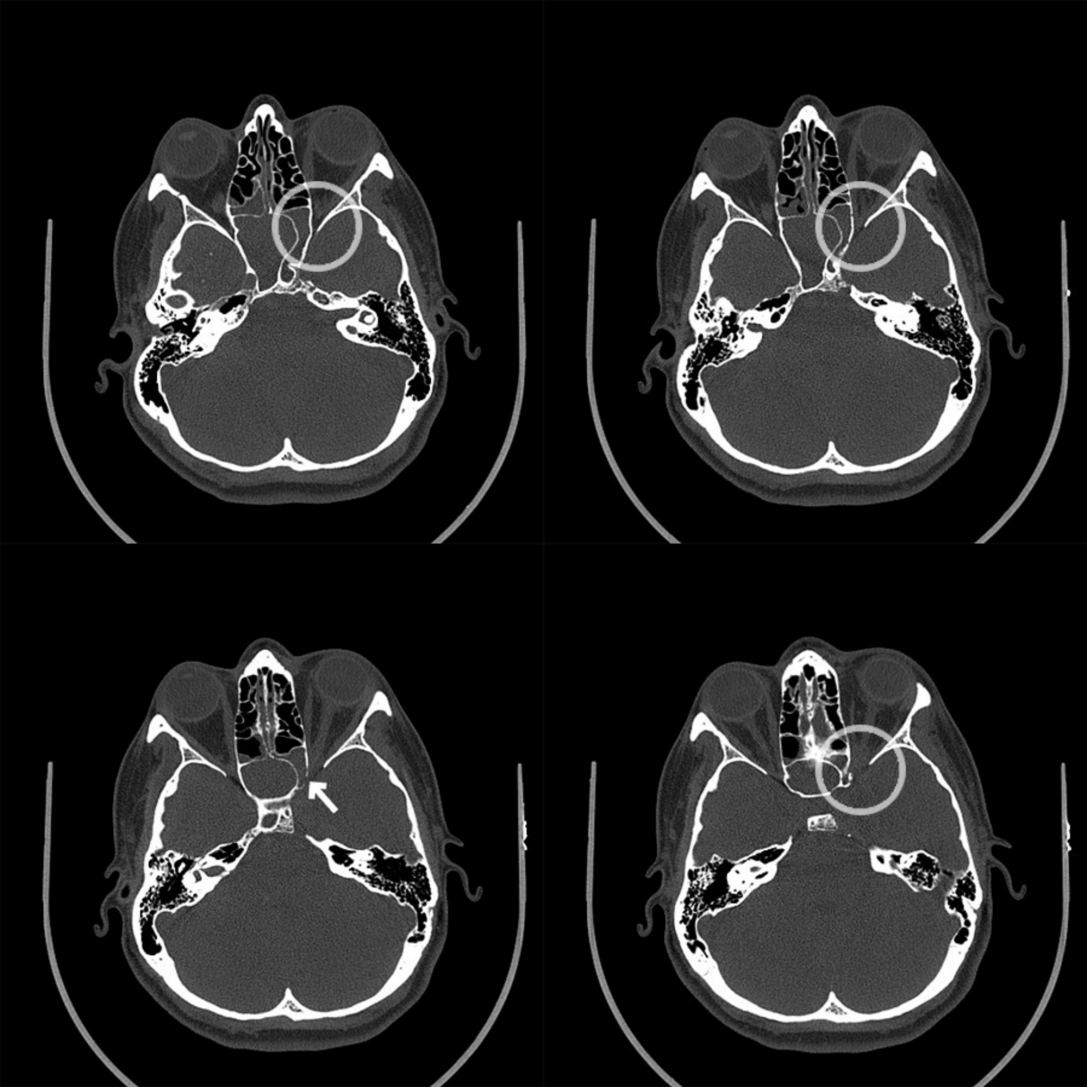
CT Images Progressive invasive sphenoid sinusitis, which invades into the left orbital apex (arrow). Crowding of the left orbital apex demonstrated for which compression of the left optic nerve cannot be excluded. Progressive bone invasion with associated osteomyelitis involving the left anterior clinoid process and the roof of the left orbit with suspicion of adjacent dural thickening effacing the suprasellar cistern, suggesting intracranial extension with associated meningitis. CT: computed tomography

Based on this new development, an emergent CT scan of the head was ordered, which revealed rapidly progressive sinusitis that eroded through the left orbital wall, causing an inflammation of the left orbital apex with radiographic evidence of meningitis concerning for a possible fungal infection (Figure 1).

Once the diagnosis of rapidly progressive sinusitis with CNS involvement (radiographically evident meningitis with a later confirmed epidural abscess) was made, the patient was started on intravenous (IV) vancomycin, 25 mg/kg, rocephin, 2 g, and amphotericin B, 5 mg/kg, concurrently. After ED intervention, the patient was emergently transferred to a tertiary care facility for evaluation by otolaryngology/ear, nose, and throat (ENT), and neurosurgery.

On arrival to the neurosurgical intensive care unit (ICU) of the tertiary care facility, the patient was evaluated by ENT and taken immediately to the operating room for left total ethmoidectomy and bilateral sphenoidotomy with the removal of contents. Cultures were obtained at the time of surgery, which grew methicillin-resistant *Staphylococcus aureus*. The patient continued with IV antibiotics, physical therapy, and occupational therapy and was discharged to a rehabilitation facility.

**Figure 2:**
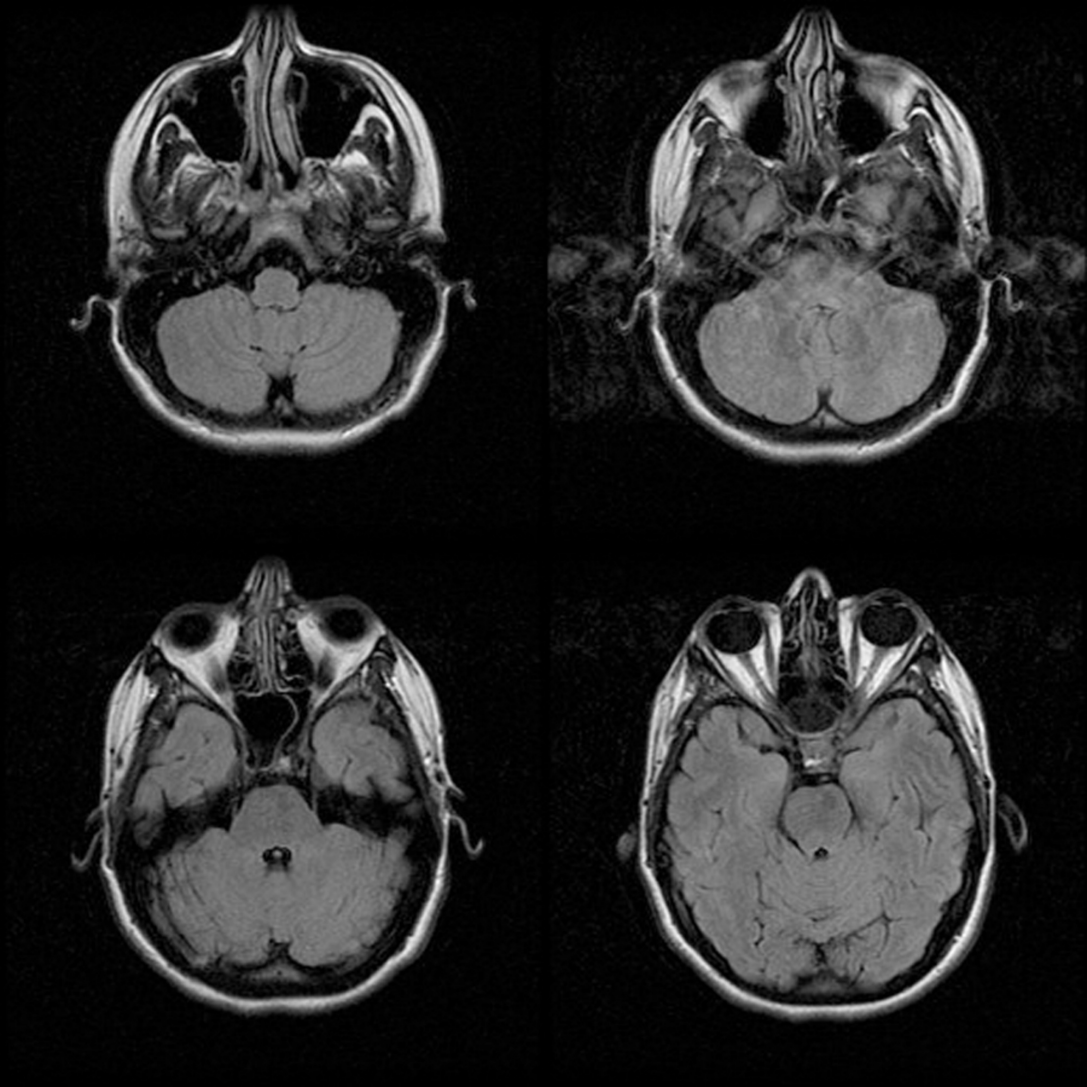
MRI Images Axial images, T2 weighted with Flair. "There has been near resolution of the mucosal disease within the paranasal sinuses with only minimal mucosal thickening remaining within the sphenoid sinuses and ethmoid air cells bilaterally. Significant improvement in the intracranial extent of disease. There has been significant improvement in the degree of inflammatory leptomeningeal enhancement with mild residual enhancement remaining within the suprasellar cistern.” MRI: magnetic resonance imaging; Flair: fluid attenuated inversion recovery

A week after admission, the patient suffered a left basal ganglia stroke, which was thought to have been caused by infective arteritis. She initially struggled with expressive aphasia but rapidly improved after the initiation of steroids and aggressive rehabilitation. At the time of discharge from the rehabilitation facility, the patient had a near-complete resolution of her mucosal disease (sinusitis), a complete resolution of her epidural abscess, and significant improvement in her leptomeningeal inflammatory enhancement on MRI (Figure 2). Additionally, the patient had a full return of speech and could perform all activities of daily living. However, walking still required supervision but difficulty was only noted with uneven surfaces.

## Discussion

The emergency department is a challenging environment for providers, as there is an inordinate amount of opportunities for errors to occur. Of note, the implementation of ED "metrics," which measures the speed and efficiency of ED providers, has pushed ED providers to work faster, with fewer resources. This push has forced providers to use primarily heuristic systems of thought in the management of patients in the ED. Heuristic systems of thought, which as mentioned previously, are prone to cognitive errors due to mental shortcuts, including pattern recognition and practice experience [5]. In order to deliver high-quality patient care and reduce the risk of negative outcomes, the following learning points should be considered. Many cognitive errors can be avoided by gathering sufficient information and developing a differential diagnosis. Consider identifying the three most life-threatening and the three most common diagnoses in addition to identifying any “red flag” symptoms for diagnoses that are “can’t miss,” i.e., emergently life-threatening – ectopic pregnancy, myocardial infarction, and subarachnoid hemorrhage. Some of the common errors seen within the emergency department include the following [2]:

Premature Closure: Uncritical acceptance of the initial diagnosis and failing to search for information to challenge the provisional diagnosis or to consider other diagnoses. By challenging the diagnosis of "migraine headache" and opening the differential diagnosis to include intracranial abnormalities, a premature closure was avoided. This refusal to accept the provisional diagnosis and maintain an open differential is how to avoid premature closure.

Anchoring: Focusing on one particular symptom, sign, piece of information, or a particular diagnosis early in the diagnostic process and failing to make any adjustments for other possibilities – either by discounting or ignoring them. Initially, anchoring can be seen when the alteration of consciousness was attributed to the use of prochlorperazine and diphenhydramine. By discounting/ minimalizing the altered mental status and anchoring on the diagnosis of migraine, a delay was observed. Any change in a patients condition offers new diagnostic information. Providers must always be open to incorporating new information into their diagnostic algorithms to avoid anchoring on a potentially incorrect diagnosis.

Diagnosis Momentum – aka Bandwagon Effect: Diagnostic labels may stick to a patient. If everyone else thinks it, it must be right! Arguably, diagnosis momentum is the most obvious error apparent in the described case. The patient was given a label of migraine headaches (potentially correctly as several, previous, thorough work-ups revealed no abnormalities) and subsequent providers worked based on the previously established diagnostic label. Each encounter with a patient must be treated as unique; the establishment of a previous diagnosis does not preclude the patient from having another diagnosis.

Additionally, another source of error to navigate in the emergency department are provider handoffs. Handoffs are an inherently unavoidable challenge presented to emergency medicine providers due to the shift style work used to staff emergency departments. ACEP recognizes the dangers of provider hand-offs and has proposed the “Safer Sign Out Protocol” in order to create a standardized approach to provider hand-offs. ACEP (via the Quality Improvement & Patient Safety section), in conjunction with the Joint Commission, expert consensus, and clinician feedback has developed the following sign-out protocol to formalize the sign-out process for ED physicians that focuses on areas that are high risk for errors [4]:

Five key steps – 5 Rs

1.     Record – Patient and essential data/updates/pending items

2.    Review – Sign-out form and computer data

3.    Round – Bedside, together

4.    Relay to the Team – Inform the nurse/team

5.    Receive Feedback – Clinical outcome

By utilizing ACEP's Safer Sign Out Protocol, the patient's altered mental status may have been noted earlier in the diagnostic process. The impact of this delay in diagnosis may not be easily characterized in this particular patient, but several diagnoses made in the ED are time sensitive and potentially life-changing. The potential to avoid a life-changing delay in patient care should make the bedside handoff process an integral piece of patient handoffs in order to maintain high-quality patient care and ensure patient safety.* *

We recommend using ACEP's “Safer Sign Out Protocol” or to create a standardized approach to patient-care transitions within the emergency department to avoid high-risk medical errors. Lastly, research has found that verbal communication with note-taking style handoffs had high rates of data loss, whereas a written form of communication with a verbal exchange (as recommended by ACEP) is associated with minimal data loss. By performing the actions described above and keeping the most common cognitive errors in mind, many of the risks associated with cognitive errors and provider hand-offs can be avoided.

## Conclusions

By avoiding cognitive errors, including premature closure, anchoring, and diagnosis momentum, the patient in the case described above was admitted, treated aggressively, and eventually discharged from the hospital after a short stay in rehabilitation with an expected full recovery. She did, however, likely experience a delay in diagnosis due to an inefficient, incomplete handoff process.
